# Evidence for the Existence of Mating Subtypes Within the *Schizophyllum commune*: Mating Behavior and Genetic Divergence

**DOI:** 10.3390/jof11040277

**Published:** 2025-04-01

**Authors:** Chen Chu, Dongxu Li, Linqing Gu, Sihai Yang, Changhong Liu

**Affiliations:** State Key Laboratory of Pharmaceutical Biotechnology, School of Life Sciences, Nanjing University, Nanjing 210023, China; mg1930112@smail.nju.edu.cn (C.C.); laolang_2012@163.com (D.L.); linqinggu01@163.com (L.G.)

**Keywords:** fungi, fungal genetics, genetic diversity, mating type, tetrapolar mating system

## Abstract

*Schizophyllum commune*, a Basidiomycota fungus with a tetrapolar mating system, serves as a key model for studying sexual reproduction. In this study, two distinct mating subtypes (I and II) were identified in strain 20R-7-ZF01, isolated from subseafloor sediment, which exhibited eight different mating interaction phenotypes. Intra-subtypes exhibited colony-symmetric tetrapolar interactions (G1), whereas inter-subtype crosses yielded colony-asymmetric phenotypes (G2) and a reduced number of fruiting bodies. Nuclear migration analysis revealed that both subtypes follow the same sexual reproductive process, suggesting functional similarities despite the different reproductive outcomes. Gene silencing of mating-type loci identified the genes *bbp2-9* and *bbp2-7* within the B locus as key factors in determining mating subtype identity. Additionally, a similar pattern of mating subtype differentiation was observed in five other *S. commune* strains from both subseafloor and terrestrial environments. These findings highlight the genetic diversity within *S. commune*, challenge the classical understanding of fungal mating systems, and provide new insights into the genetic evolutionary mechanisms governing fungi with tetrapolar mating systems.

## 1. Introduction

*Schizophyllum commune* is a widely distributed white-rot fungus [1], classified within the genus *Schizophyllum*, family Schizophyllaceae, order Agaricales, class Agaricomycetes, and phylum Basidiomycota. *S. commune* valued for their ability to produce valuable metabolites, among which Schizophyllum polysaccharides are involved in immunomodulation, antitumor activity, and potential wound healing [2]; the bioactive compounds produced can also have antioxidant and anti-inflammatory effects by neutralizing free radicals and reducing oxidative stress [3]. At the same time, *S. commune*, with its quadrupolar mating system and short life cycle (10–14 days), are ideal model organisms for exploring these complex aspects of fungal biology [4,5,6]. The life cycle of *S. commune* begins with the germination of meiospores, which form monokaryotic mycelium. When compatible mating types meet, these monokaryotic strains can fuse to form fertile dikaryons. This fusion process is marked by the development of clamp connections [7], and culminates in the formation of fruiting bodies, where karyogamy and meiosis take place, producing basidiospores that can germinate into new monokaryotic mycelia [8].

The genetic basis of sexual reproduction in *S. commune* has been the focus of research for over a century. In 1920, Kniep first proposed that sexual reproduction in *S. commune* involves multiple incompatible alleles located on two independent genetic loci [9]. These loci, known as the A and B loci (also referred to as A and B loci factors), are located on separate chromosomes [1]. Each locus consists of two linked subloci (Aα, Aβ, Bα, and Bβ). Through extensive sampling and labeling of mating-compatible subloci, Raper et al. [10] estimated that *S. commune* has approximately nine specificities at Aα (e.g., Aα1, Aα2, Aα3... Aα9), about 32 at Aβ, and nine at both Bα and Bβ. This diversity results in an estimated 23,328 possible mating types [11]. At the molecular level, the A locus encodes two types of homeodomain (HD) transcription factors, HD1 and HD2. In contrast, the B locus contains pheromone (Ph) and pheromone receptor (PR) genes [12,13], collectively known as mating-type (MAT) genes. A substantial number of mating-type alleles are harbored at these loci [14], with natural populations containing up to 288 alleles at the A locus and 81 at the B locus [15]. The extensive segregation of A and B alleles within the same *S. commune* population also contributes to its remarkable genetic diversity [16].

In the sexual reproduction of *S. commune*, the A and B factors play key roles. The A factor governs processes such as clamp connection formation and nuclear pairing, while the B factor is involved in regulating hook cell dynamics and nuclear migration [17]. During sexual reproduction, the HD genes at the A locus use an N-terminal dimerization motif to form heterodimers between the HD1 and HD2 specificities, which are essential for activating the A-specific signaling pathway [18]. On the other hand, the *Ph/PR* genes trigger the B-specific signaling cascade, where a single receptor can recognize multiple non-self-pheromones [19]. Notably, nuclear exchange between monokaryotic strains occurs only when the pheromone genes of one strain are recognized by the pheromone receptor genes of another [20]. The mating system of *S. commune* is highly representative in the fungal world, providing insight into issues such as the evolution of fungal mating systems, mechanisms for maintaining diversity, and population genetics. By studying the mating system of *S. commune*, we can gain a deeper understanding of how fungi promote heterokaryosis through complex genetic mechanisms, thereby increasing genetic diversity and adaptability. This complex mating system makes *S. commune* an excellent model for studying the genetic mechanisms underlying sexual reproduction and genetic divergence in fungi.

To facilitate the expression and identification of mating types, Raper et al. [21] utilized “x” and “y” to label the two distinct sets of A and B factors in dikaryotic strains, resulting in genotypes AxAyBxBy. Four potential mating genotypes emerge during sexual reproduction: AxBx, AxBy, AyBx, and AyBy. Among these, only the AxBx and AyBy or the AxBy and AyBx mating combinations lead to the formation of fruiting bodies, thus completing the entire sexual life cycle. This tetrapolar mating system generates four types of mating interactions: (1) A≠B≠ (both factors differ), (2) A=B= (both factors are the same), (3) A≠B= (A differs, B is the same), and (4) A=B≠ (A is the same, B differs) [22]. Nuclear migration occurs at the mycelial junction between strains that exhibit different B factors (B≠) [23,24], emphasizing the crucial role these interaction phenotypes play in determining mating compatibility.

Traditional methods for determining mating types in tetrapolar fungi have typically relied on examining colony morphology based on three mating reactions: mycelial compatibility (A≠B≠), barrage reaction (A≠B=), and flat reaction (A=B≠) [25,26]. It is generally assumed that the A=B= reaction, often overlooked, produces symmetrical mating interaction phenotypes when co-cultured. However, it has remained uncertain whether any differentiation exists within these mating phenotypes, as both symmetric and asymmetric colony growth patterns have been observed in A≠B= pairings of *S. commune* [25,27]. Currently, there are no reports on whether mating subtypes exist under the same mating type. Most previous studies have focused solely on the “mating type division” of these two loci, that is, identifying the combination of A and B locus alleles to determine the “mating type”, while overlooking the question of whether there is further differentiation within the same mating type. This makes it difficult for us to distinguish subtypes or differentiated groups under the same mating type, limiting our in-depth understanding of mating type diversity and its evolutionary process. Therefore, the study of mating subtypes of Schizophyllum is not only necessary to fill the gaps in previous research, but also provides a theoretical basis for the discovery of mating subtypes in other fungi. It is of great significance to understand the relationship of fungi, the evolutionary process of homothallic and heterothallic mating, and the evolution and origin of fungal groups.

In this study, we made a significant discovery: a dikaryotic strain of *S. commune* (20R-7-ZF01), isolated from ~20-million-year-old coal-bearing sediments [28,29], displayed unexpected genetic differentiation. From the same fruiting body, we obtained 149 monokaryotic basidiospore strains. Cross-mating these strains revealed eight distinct mating phenotypes, which we categorized into two groups: G1 (symmetrical colony growth) and G2 (asymmetrical colony growth), with four phenotypes in each group. The ratio of G1 to G2 strains was 1:1, indicating a clear differentiation in mating types within *S. commune*. Based on these findings, we propose the existence of mating subtypes within *S. commune* and provide experimental evidence to support this novel observation. Our research not only deepens the understanding of the genetic mechanisms driving mating type differentiation in basidiomycetes but also sheds new light on genetic differentiation processes in tetrapolar fungi, contributing to advancing the broader understanding of fungal genetics, particularly regarding mating-type systems, and laying the foundation for future studies in the field.

## 2. Materials and Methods

### 2.1. Fungal Strains

This study utilized six pure cultured dikaryotic strains of *Schizophyllum commune*. Four of these strains (20R-7-ZF01, 15R-5-ZF01, 6R-2-ZF01, and 24R-3-ZF01) were previously isolated from coal-bearing sediment samples approximately 2 km beneath the seafloor of the Shimokita Peninsula, Japan [29]. The CFCC-85778 and CFCC-86616 strains, originally isolated from terrestrial environments of *Catalpa bungei* C. A. Mey in Liaoning Kuandian and *Amygdalus persica* L. in Shandong Laiwu, respectively, were obtained from the China Forestry Culture Collection Center. To distinguish between mono- and dikaryotic strains, the dikaryotic strains in this study were labeled as “xx-ZF-xx” (e.g., 20R-7-ZF01). In contrast, the monokaryotic strains were labeled as “xx-F-xx” (e.g., 20R-7-F01). The monokaryotic strain 20R-7-F01 [30] was derived from the dikaryotic strain 20R-7-ZF01 and served as a DNA donor for nuclear migration fluorescence experiments. All strains were stored on MM [2] slants at 4 °C and reactivated every three months.

### 2.2. Obtaining Monokaryotic Strains

To obtain monokaryotic strains of *S. commune*, cultures were initially inoculated into sterile bottles containing a modified substrate mixture composed of 30% oak sawdust, 60% cottonseed hulls, 10% wheat bran, 1% sucrose, 1% gypsum, and 50–60% water [31]. The inoculated bottles were incubated in the dark at 30 °C and 60% humidity for approximately 7 days to promote mycelial growth. Following this incubation, the bottles were transferred to a well-lit environment at 25 °C to induce the formation of mature fruiting bodies.

**Figure 1:**
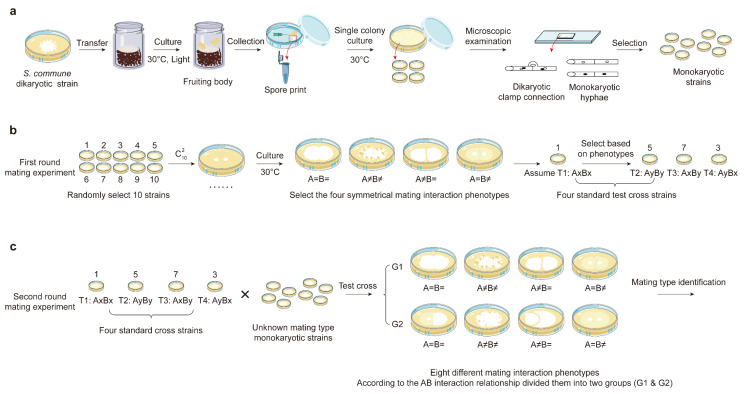
Flowchart of *S. commune* mating experiments. (**a**) Isolation of monokaryotic cultures derived from basidiospores. (**b**) Confirmation of mating tests using standard cross strains. (**c**) Identification of mating types.

To isolate single-spore cultures (monokaryons), a mature fruiting body was harvested and placed in a Petri dish at room temperature for 1–2 days, allowing spores to be released. The released spores were then rinsed with 1 mL of sterile water to create a spore suspension. This suspension was diluted 10^4^–10^5^ times with sterile water, and a 0.2 mL dilution was plated onto a Potato Dextrose Agar (PDA) medium. The plates were incubated at 30 °C for three days with air humidity maintained at 80% to allow spore germination. Mycelial growth from individual germinating spores was carefully picked using a fine needle, ensuring that the mycelium exhibited no clamp connections, which confirmed the monokaryotic nature of the culture. These monokaryotic strains were then used for subsequent mating experiments (Figure 1a).

### 2.3. Mating Type Identification

To identify the mating types of *S. commune*, a dual-culture technique was employed using 10 randomly selected monokaryotic cultures derived from the same dikaryotic parent. Mycelial plugs (6 mm in diameter) containing actively growing mycelium were placed 10 mm apart on a minimal medium (MM) in 60 mm Petri dishes and incubated at 30 °C for 7 days. Mating interactions at the contact zones were observed, with compatibility determined by the presence or absence of clamp connections. Based on the phenotypic outcomes at the contact zones, four classical mating interaction phenotypes were identified: A=B=, A≠B≠, A≠B=, and A=B≠, as described by Darmono and Burdsall [32].

Subsequently, one monokaryotic culture was randomly selected and designated as mating type AxBx, serving as a reference for determining the mating types of the remaining nine cultures. Using the dual-culture technique, mating interactions between the reference strain and other monokaryons of the same parent were assessed, yielding four distinct mating genotypes: AxBx, AyBy, AxBy, and AyBx (Figure 1b).

To further characterize the mating behavior, one strain from each of the four mating genotypes was co-cultivated with monokaryotic strains that were isolated from the fruiting bodies. This co-cultivation resulted in two groups (G1 and G2) of mating reaction phenotypes, each exhibiting four classical mating interaction phenotypes (Figure 1c). The ratios of the four mating types among the isolated monokaryons were statistically analyzed using the chi-squared test (χ^2^) as described by Li et al. [26]. This approach provided a comprehensive method for identifying and categorizing the mating types of *S. commune*.

### 2.4. Histone-Assisted Merged Fluorescence (HAMF) Assays

To investigate hyphal fusion and nuclear migration events, we constructed four plasmids: pGH2A, pRH2A, pCH2A, and pYH2A, each containing the *H2A* gene (PV339484) (amplified by PCR from the *S. commune* strain 20R-7-F01), and fused to different fluorescent proteins. These plasmids, based on pIG1783, were developed following the protocols outlined by Rech et al. [33]. Specifically, pGH2A encodes the H2A-EGFP fusion protein, while pRH2A, pCH2A, and pYH2A encode the H2A-DsRed, H2A-ECFP, and H2A-EYFP fusion proteins, respectively. The PCR primers used are listed in Table S1.

Each plasmid was individually transformed into specific mating-type strains using PEG-mediated transformation, as described by Van Peer et al. [34]. The corresponding transformation plasmids for mating subtype strains are shown in Table S2.

The transformed strains were then used in HAMF assays, in which two strains, each harboring a different plasmid, were co-inoculated at separate spots on an MM solid medium, spaced 4 cm apart. After incubating the plates at 30 °C for 3 days, mycelia from the contact zones of both strains were examined for hyphal fusion and nuclear migration using an LSM980 (Zeiss, Jena, Germany) two-photon confocal microscope. The acquired images were processed and analyzed using ZEISS ZEN 3.1 (blue edition) [35]. This experimental scheme enabled detailed visualization and analysis of hyphal fusion and nuclear dynamics during mating and fusion events.

### 2.5. Gene Silencing of Pheromone-Encoding Genes bbp2-9 and bbp2-7 in S. commune

To investigate the roles of the pheromone-encoding genes *bbp2-9* (PV339486) and *bbp2-7* (PV339485) in the development of mating subtypes in *S. commune*, we introduced the plasmids pLKO.1-EGFP-bbp2-9 and pLKO.1-EGFP-bbp2-7 into *S. commune* strains representing the AxBx subtype I strain (S4y) and AyBy subtype I strain (S5y), respectively. Plasmid construction and transfection were performed using PEG-mediated protoplast transformation, as described by Holzberg et al. [36]. We used online short hairpin RNA (shRNA) design tools (https://www.vectorbuilder.cn/tool/shrna-target-design.html) (accessed on 20 June 2023) to design shRNAs targeting the *bbp2-9* and *bbp2-7* genes. The sequences of the shRNAs are provided in Table S3. This gene silencing strategy allowed us to examine the role of these pheromone-encoding genes in the formation of mating subtypes in *S. commune*.

### 2.6. Genome Sequencing and Mating-Type Gene Annotation

Genomic DNA was extracted from *S. commune* strains representing each of the identified mating subtypes (eight strains in total). The DNA was then fragmented to construct a sequencing library with an average insert size of approximately 500 bp. Next-generation sequencing (NGS) was performed on the Illumina NovaSeq PE150 platform at APRxBIO (Shanghai, China). The raw sequencing data were initially processed using FASTQC software (version 0.12.1) to filter out adapters and low-quality reads (Q < 20) [37]. The clean data were then assembled into scaffold-level contigs using assembly software SPAdes (version 3.12.0) [38]. A draft genome was generated after gap closure using GapCloser (version 1.12), and the integrity of the genome assembly was assessed using BUSCO software (version 3.0.2) [39]. The assembly quality was further evaluated using Quast software (version 5.3.0) [40].

To annotate mating-type genes, we performed BLASTP (version 2.2.26) homology searches against the GeneBank non-redundant database [41] using the methods described by Ohm et al. [1] and Wirth et al. [42]. Our analysis focused on key genetic markers, including the MATA homeodomain (*HD*) genes, MATB pheromone (*Ph*) genes, and pheromone receptor (*PR*) genes. The extent of the A mating-type loci in *S. commune* was determined by examining the mitochondrial intermediate peptidase gene (*mip*) and the β-flanking genes (*β-fg*). For the B mating-type loci, the extent was defined by the presence of genes encoding pheromone-like receptors, including *brl1*, *brl2*, and *brl3*, located on either side of the B*α* and B*β* subloci. These annotations provided a comprehensive understanding of the mating-type loci within the *S. commune* genome, facilitating the identification and characterization of genes involved in its complex mating system.

## 3. Results

### 3.1. Isolation and Characterization of Mating Subtype Strains in S. commune

A total of 149 monokaryotic strains were isolated from the fruiting bodies of the subseafloor fungus *S. commune* strain 20R-7-ZF01. Genetic analysis identified four distinct mating genotypes: AxBx, AyBy, AxBy, and AyBx. Specifically, 42 strains exhibited the AxBx genotype, 38 strains were of genotype AyBy, another 38 strains were AxBy, and 31 strains displayed AyBx. A chi-square test (χ^2^ = 1.685, *p* > 0.05) revealed no significant difference in the distribution of these genotypes, suggesting that the distribution follows Mendelian inheritance patterns and supports the conclusion that strain 20R-7-ZF01 exhibits a typical tetrapolar heterothallic mating system.

**Figure 2:**
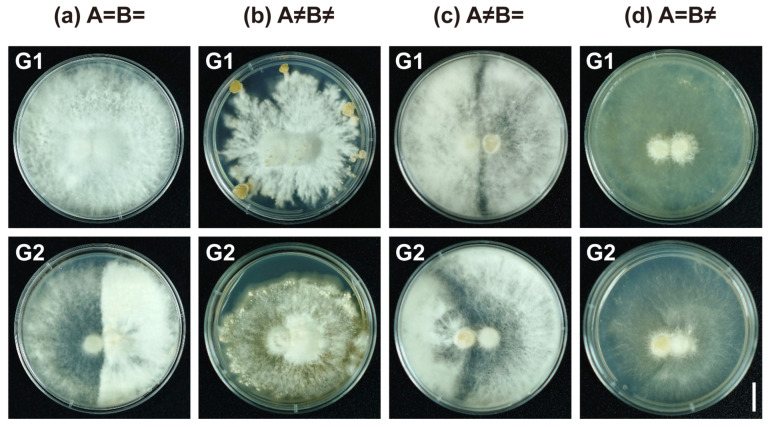
Tetrapolar mating interactions of subseafloor *S. commune* 20R-7-ZF01. (**a**) Common-AB pairings show an “indistinguishable” reaction; (**b**) Compatible pairings exhibit a “compatible” reaction; (**c**) Common-B pairings display a “barrage” reaction; (**d**) Common-B pairings show a “flat” reaction with lack of aerial mycelium. Differentiation of responses during mating, as per Kothe’s method [27]. Mating interaction phenotypes were categorized into two groups, G1 and G2, as detailed in Table 1; G1 includes symmetric phenotypes, while G2 comprises asymmetric phenotypes. Bar = 1 cm.

To further investigate the mating types, we performed two rounds of dual-culture mating experiments using the method described by Niederpruem et al. [25]. A total of 596 pairwise combinations of the 149 strains were tested (Figure 1). The results revealed eight distinct mating reaction phenotypes, which were grouped into two main categories (G1 and G2), each containing four mating reaction types: A=B=, A≠B≠, A≠B=, and A=B≠ (Figure 2). The G1 phenotype exhibited nearly identical hyphae phenotypes on both sides, while the G2 phenotype showed an asymmetrical relationship. The detailed classification criteria are outlined in Table 1. These mating phenotypes corresponded to the four mating genotypes (AxBx, AyBy, AxBy, and AyBx). The genotypes within G1 were labeled as Subtype I (AxBx-I, AyBy-I, AxBy-I, AyBx-I), while the genotypes in G2 were classified as Subtype II (AxBx-II, AyBy-II, AxBy-II, AyBx-II).

**Figure 3:**
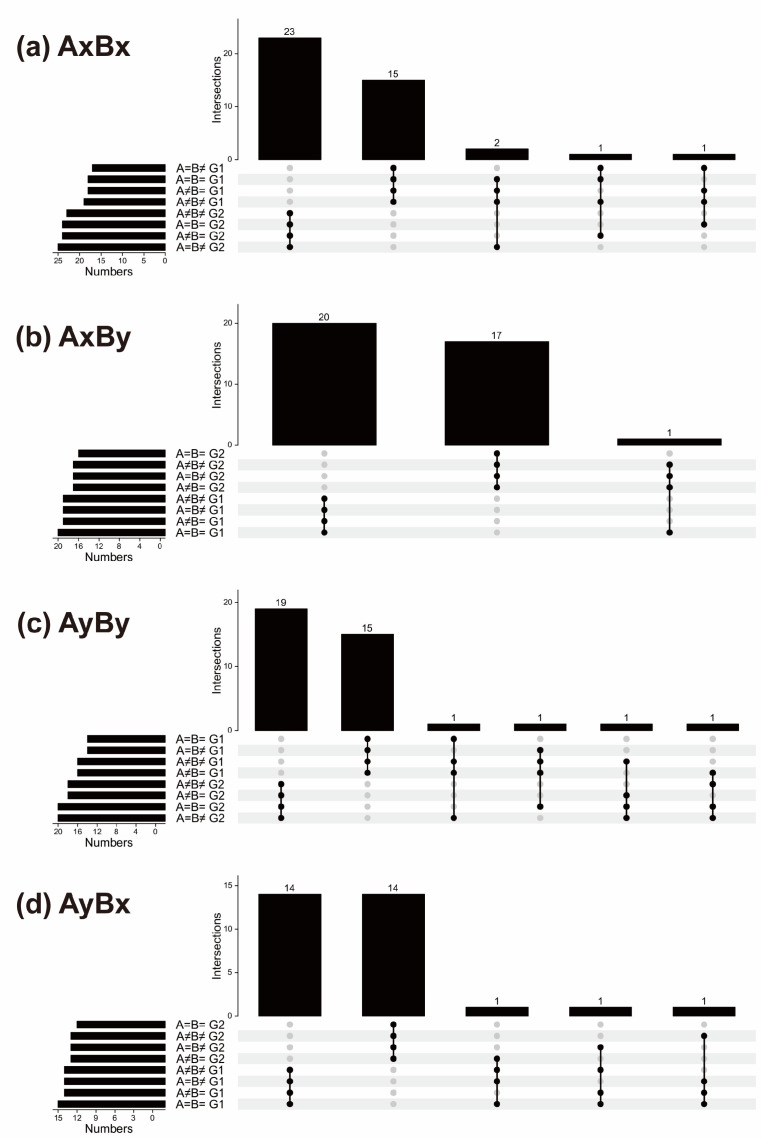
UpSet plots of mating interaction phenotypic comparisons in monokaryotic strains from *S. commune* 20R-7-ZF01. Comparisons were made separately according to the different mating types: (**a**) AxBx; (**b**) AxBy; (**c**) AyBy; and (**d**) AyBx. The UpSet plot functions similarly to a Venn diagram but is more effective in handling and visualizing data from a larger number of groups [43]. The bars below the left side of the *x*-axis represent the total number of strains in each group, corresponding to the set size. The dot plots below the right side of the *x*-axis show the strain intersection zones, and indicate which groups are represented in the histogram above. Black means that this point has data, and gray means that this point has no data. The histogram above shows the number of strains represented by this group.

Statistical analysis of the mating reaction phenotypes between G1 and G2 indicated a near 1:1 ratio (73:76), although a slight difference in the distribution of mating genotypes was observed (Figure 3). Within G1, the distribution of AxBx-I, AxBy-I, AyBy-I, and AyBx-I was 15, 20, 15, and 14, respectively, representing 23.4%, 31.3%, 23.4%, and 21.9% of the total 64 analyzed strains. In G2, the distribution of AxBx-II, AxBy-II, AyBy-II, and AyBx-II was 23, 17, 19, and 14, respectively, accounting for 31.5%, 23.3%, 26.0%, and 19.2% of the 73 strains. This disparity in the mating genotype distribution between G1 and G2 highlights the complexity of the mating system in *S. commune*.

**Figure 4:**
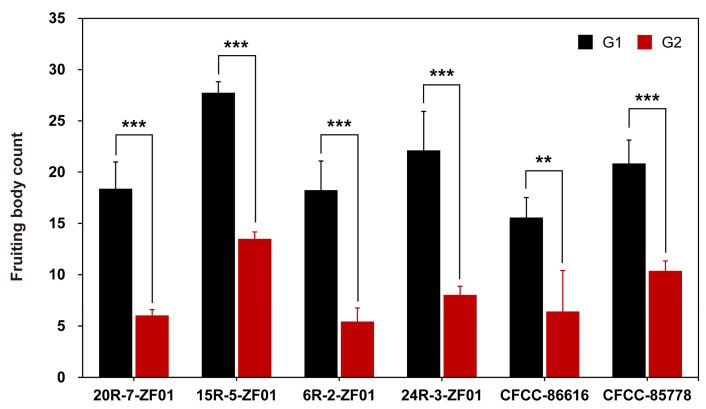
Mean number of fruiting bodies in six *S. commune* dikaryotic strains (A≠B≠ interaction). The black bars represent phenotypes from Group 1 (G1) with A≠B≠ interactions, while red bars represent phenotypes from Group 2 (G2). Values are mean ± S.E. Statistical significance is indicated as follows: *** *p* < 0.001, ** *p* < 0.01 (*n >* 3).

Further morphological analysis revealed distinct differences between the mating genotypes. In single cultures, strains from both G1 and G2 exhibited similar colony morphologies (Figure S1). However, during dual-culture experiments, the A≠B≠ phenotype in G1 demonstrated significantly more robust hyphal growth and higher fruiting body production compared to the G2 strains. The G2 strains exhibited finer hyphae, fewer fruiting bodies, and a reduced competitive ability (Figure 2 and Figure 4). Microscopic examination of the mating contact zones (Figure S2) confirmed these findings. In G1, strains exhibited greater symmetry in hyphal morphology at the contact zone, characterized by consistent septal formation and well-formed clamp connections, which are associated with the A≠B≠ phenotype. In contrast, G2 strains exhibited variability in septal spacing, hyphal thickness, branching patterns, and the formation of clamp connections.

**Figure 5:**
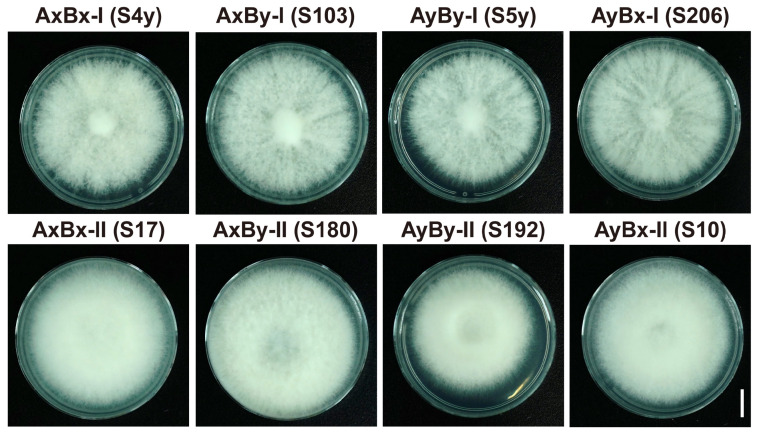
Tetrapolar mating subtype monokaryotic strains of *S. commune* 20R-7-ZF01. The strains representing each mating subtype are indicated in the parentheses. Bar = 1 cm.

To further validate the existence of distinct mating subtypes in *S. commune*, we performed intra-subtype (I × I or II × II) and inter-subtype (I × II) mating experiments using eight representative strains from different mating subtypes (Figure 5). The results from 36 mating sets revealed that the intra-subtype mating consistently produced G1 mating reaction phenotypes, while inter-subtype mating resulted in G2 phenotypes (Table 2). This confirmed the regularity of differences in interaction phenotypes between Subtypes I and II.

Additionally, we examined the mating type composition of three dikaryotic strains from subseafloor sediment samples (6R-2-ZF01, 15R-5-ZF01, and 24R-3-ZF01) and two from terrestrial environments (CFCC-85778 and CFCC-86616). The results were consistent with those observed for the 20R-7-ZF01 strain, with each of the five strains displaying two sets of eight mating reaction phenotypes (Figure S3). This reinforces the conclusion that *S. commune* indeed exhibits two distinct mating subtypes.

### 3.2. Observation of Hyphal Fusion and Nuclear Migration During Mating in S. commune Using Fluorescently Labeled Recombinant Strains

To investigate hyphal fusion and nuclear migration during mating, we generated eight recombinant strains, each representing one of the eight mating subtypes of *S. commune* (Figure 5). These strains were engineered with plasmids pGH2A, pRH2A, pCH2A, or pYH2A, which express fluorescently tagged histone H2A proteins (Table S2). Dual-culture mating experiments were conducted using these fluorescent strains, and HAMF assays were employed to monitor hyphal fusion and nuclear migration. This approach allowed us to observe both intra-subtype and inter-subtype mating events.

**Figure 6:**
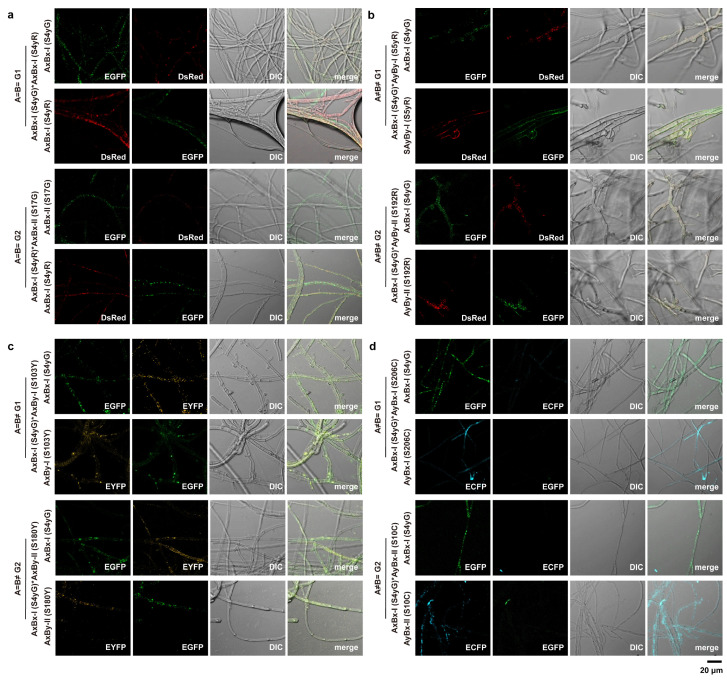
Fluorescence microscopy visualization of interactions between mating subtype strains. (**a**) A=B= interaction; (**b**) A≠B≠ interaction; (**c**) A=B≠ interaction; and (**d**) A≠B= interaction, showing both intra- (i.e., I × I or II × II) and inter- (i.e., I × II) mating subtype strains. The micrographs display fluorescence images, DIC images, and merged images of mycelium on both sides of the mating interaction, with the sampled strains labeled on the left side of each row. The subseafloor strains representing each mating subtype are indicated in parentheses. “G”, “R”, “Y”, and “C” represent strains transfected with EGFP, DsRed, ECFP, and EYFP fluorescent markers, respectively. * Indicates that the strains are in a mating relationship. Bar = 20 μm.

Our observations revealed that strains from different mating subtypes displayed similar patterns of hyphal fusion, nuclear migration, and nuclear fusion (Figure 6 and Figure S4, Table 3). These findings suggest that, despite the distinct classification of mating subtypes, the fundamental processes of hyphal fusion and nuclear migration are conserved across the different mating types of *S. commune*. This highlights the shared underlying mechanisms of mating behaviors, regardless of subtype differences.

### 3.3. Identification of Putative Mating-Type Loci in S. commune and Their Role in Mating Subtype Formation

**Figure 7:**
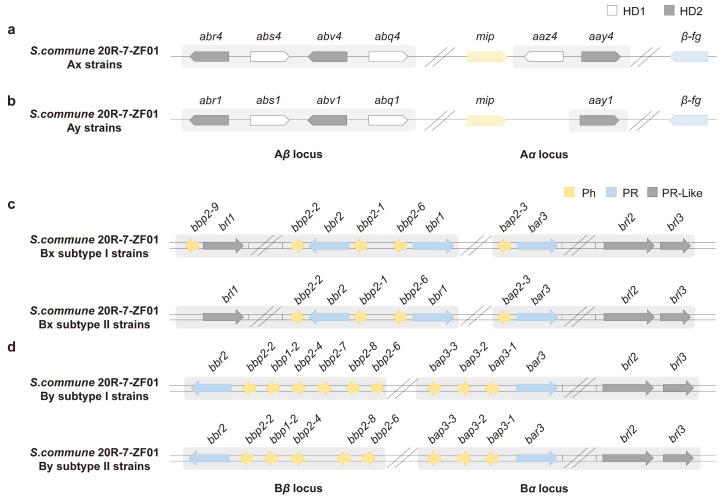
Putative matA and matB loci in *S. commune* monokaryotic strains. (**a**) Genes encoding HD1 and HD2 homeodomain proteins in the Ax mating type. (**b**) Genes encoding HD1 and HD2 homeodomain proteins in the Ay mating type. (**c**) Genes encoding Ph and PR proteins in Bx mating subtypes I and II. (**d**) Genes encoding Ph and PR proteins in By mating subtypes I and II. Strains with the same mating type but different subtypes share the same genetic composition.

To investigate the genetic basis of mating subtype differentiation in *S. commune*, we sequenced the genomes of eight representative strains from the two mating subtypes (Figure 5). We conducted a detailed analysis of their mating-type loci. Our findings revealed that strains from both the Ax-I and Ax-II subtypes share an identical A factor, which contains three HD1-encoding genes (*abs4*, *abq4*, *aaz4*) and three HD2-encoding genes (*abr4*, *abv4*, *aay4*) located within the Aα and Aβ loci (Figure 7a, Table S4). In contrast, strains from the Ay-I and Ay-II subtypes, while carrying the same A factor, display a distinct genetic composition at the mating-type loci. These strains harbor two HD1-encoding genes (*abs1*, *abq1*) and three HD2-encoding genes (*abr1*, *abv1*, *aay1*) (Figure 7b, Table S4). Notably, the *aaz4* gene, which encodes the Z protein that interacts with the Y protein during mating organization, is present in Ax strains but absent in Ay strains. This absence highlights a significant genetic divergence between Ax and Ay strains, suggesting that the Z protein plays a key role in mating compatibility and sexual reproduction in *S. commune*.

In addition to the A mating-type loci, we also observed considerable differences in the gene composition and structure of the B mating-type loci, both between the Bx and By strains and within the Bx-I and Bx-II subtypes, as well as the By-I and By-II subtypes. The most significant genetic differences were found between Bx and By strains. Specifically, Bx strains contain 4 to 5 *Ph* genes, 3 *PR* genes, and 3 *PR-like* genes (Figure 7c, Table S5), while By strains carry 8 to 9 *Ph* genes, 2 *PR* genes, and 2 *PR-like* genes (Figure 7d, Table S5). The distinction between the Bx-I and Bx-II subtypes is characterized by the presence of an additional *Ph* gene, *bbp2-9*, in Bx-I strains (Figure 7c). Similarly, By-I and By-II subtypes differ by the presence of an extra *Ph* gene, *bbp2-7*, in By-I strains (Figure 7d). These additional *Ph* genes, particularly *bbp2-9* in Bx-I and *bbp2-7* in By-I, likely play a crucial role in determining mating subtype identity. The presence of these genes may influence mating preferences and reproductive success, contributing to the complexity and diversity of sexual reproduction in *S. commune*.

### 3.4. Impact of bbp2-9 and bbp2-7 Gene Silencing on Mating Subtype Formation

To explore the impact of silencing the *bbp2-9* and *bbp2-7* genes on mating subtype formation in *S. commune*, we introduced plasmids pLKO.1-EGFP-bbp2-9 and pLKO.1-EGFP-bbp2-7 into AxBx subtype I strain S4y and AyBy subtype I strain S5y, respectively (Figure 5). This resulted in the creation of the gene-silenced strains S4y_^Δ^*bbp2-9* and S5y_^Δ^*bbp2-7* (Figure S5).

**Figure 8:**
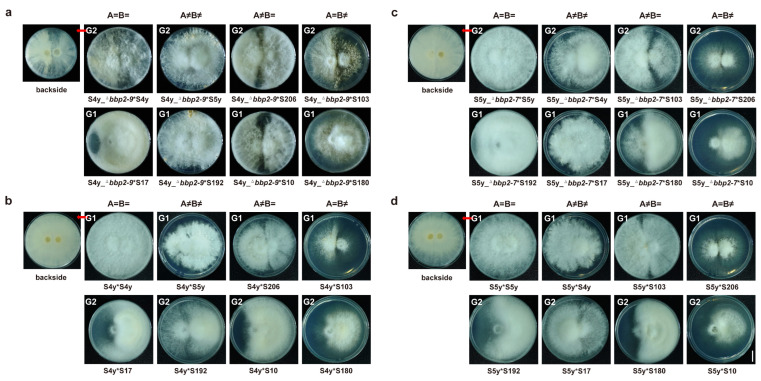
Tetrapolar mating interactions (G1 and G2) of *bbp2-9* and *bbp2-7* gene-silenced strains, S4y_^Δ^*bbp2-9* and S5y_^Δ^*bbp2-7*. (**a**) S4y_^Δ^*bbp2-9* strain; (**b**) S4y strain; (**c**) S5y_^Δ^*bbp2-7* strain; and (**d**) S5y strain showing eight distinct mating phenotypes. * Indicates that the strains are in a mating relationship. Bar = 1 cm.

Following gene silencing, we crossed these modified strains with eight different mating subtype strains (Figure 5) to assess the influence of silencing *bbp2-9* and *bbp2-7* on mating behaviors. Although the silencing did not eliminate the expression of these genes, we observed noticeable changes in mating phenotypes. Specifically, strains with *bbp2-9* silencing (S4y_^Δ^*bbp2-9*) exhibited a shift from the original G1 mating phenotype toward a G2-like mating behavior (Figure 8a,b). In contrast, strains with *bbp2-7* silencing (S5y_^Δ^*bbp2-7*) displayed a shift from the original G2 phenotype to characteristics typical of G1 mating behavior (Figure 8c,d). These findings highlight the critical role of *bbp2-9* and *bbp2-7* in determining mating subtype identities and associated phenotypes. Silencing either of these genes resulted in a reversal of mating behavior, further supporting the conclusion that these pheromone-encoding genes within the B mating-type locus are essential for the proper establishment of distinct mating subtypes in *S. commune*.

## 4. Discussion

Sexual reproduction is fundamental to species evolution, functioning as a significant driver of genetic diversity and adaptation [44]. In the fungal realm of the eukaryotic kingdom, the mating system has a profound influence on reproductive traits and evolutionary trajectories [45,46,47]. *S. commune*, well-known for its tetrapolar mating system, serves as a model organism for exploring these complex aspects of fungal biology [6]. In this study, we focused on dikaryotic strain 20R-7-ZF01, isolated from subseafloor sediment, with a particular emphasis on the mating behavior and genetic divergence of the monokaryotic strains derived from it. Our results reveal the existence of two distinct mating subtypes in *S. commune*, designated as subtypes I and II. Mating interactions occurring both within and between these subtypes can yield eight different mating interaction phenotypes (Figure S6). This finding challenges the long-standing, oversimplified perspective on the tetrapolar mating system in *S. commune*.

Traditionally, the four tetrapolar mating types (AxBx, AxBy, AyBx, and AyBy) of *S. commune* have been linked to four defined mating interactions: A=B=, A≠B≠, A=B≠, and A≠B=, each producing specific mating interaction phenotypes [22,48]. However, a significant limitation in the previously established phenograms is that the A=B= mating interaction phenotype was identified using a single colony rather than a dual-culture assay [25]. Additionally, some tetrapolar fungi identifications have been reported without the A=B= mating phenotype being observed and determined before other phenotypes are identified [26,49].

In our study, A=B= mating interactions were rigorously analyzed using a dual-culture approach, revealing a colony-asymmetry phenotype characterized by significant differences in hyphal density on both sides of the colony. This phenotype exhibits a demarcation line without an interval, and closely resembles the A≠B= phenotype. Such overlapping characteristics pose significant challenges in the accurate identification of mating phenotypes. Upon analyzing 596 phenotypes from 149 monokaryotic strains derived from the same fruiting body, we found that strains exhibiting the colony-asymmetry A=B= phenotype also displayed asymmetric A≠B≠, A=B≠, and A≠B= phenotypes (classified as G2) in mating experiments. In contrast, another group of strains consistently produced fully colony-symmetric mating interaction phenotypes (G1). Notably, these two groups appeared in a nearly 1:1 ratio, suggesting that colony symmetry is not merely an artifact of experimental error or inoculation conditions. Since the overall morphology of G2 aligns with the mating reactions of traditional tetrapolar mating strains [27], we propose the concept of mating subtypes rather than suggesting the emergence of new mating types.

In *S. commune*, the generation of mating types is governed by the A and B mating-type loci. It involves key processes such as nuclear migration, nuclear pairing, and hook cell formation [6,17]. Among these, nuclear migration is a crucial aspect of fungal sexual reproduction [24,50]. Therefore, in this study, we also explored nuclear migration during the mating of different mating subtypes using a fluorescence-based observation method, which has been proven effective for studying nuclear migration events [51,52]. By using distinct fluorescent markers, we achieved a more intuitive visualization of bidirectional nuclear migration during mating interactions. Our findings corroborated previous research: (i) in A=B= pairings, mycelial migration occurs, but no nuclear exchange takes place; (ii) in A≠B≠, bidirectional nuclear migration occurs, and clamp connections are formed; (iii) in A=B≠, bidirectional nuclear migration occurs, but clamp connections do not form; and (iv) in A≠B=, neither nuclear nor hyphal exchange occurs, and clamp connections are absent. This result is consistent with the understanding that A mating-type loci control clamp connection formation and B mating-type loci control nuclear migration [17].

It is well known that the A≠B≠ mating reaction can produce fruiting bodies after exposure to light [22,53]. However, our study showed that the number of fruiting bodies formed in the A≠B≠ of inter-subtype mating (form G2 phenotypes) and intra-subtype mating (form G1 phenotypes) is significantly different. The number of fruiting bodies in the G2 phenotypes was only 30–50% of that in the G1. A similar difference was observed in the number of clamp connections between the G1 and G2 phenotypes; the number in G2 was approximately one-third of that in the G1 phenotypes within the same field of view. Although we do not know the cause of the difference in fruiting body formation between G2 and G1, this may be linked to the expression of genes such as *TRP1*, *hom1*, *fst3*, and *fst4*, which are known to play critical roles in regulating fruiting body formation [54,55]. It may also be attributed to mitochondrial–nuclear incompatibility, similar to the phenomenon reported by Moran et al. [56], or to downstream activation of vegetative incompatibility, as observed by Ament-Velásquez et al. [57] in *Podospora anserina*. Further transcriptomic and in-depth analyses will be necessary to fully elucidate this phenomenon.

Since the A and B mating-type loci control the mating type in *S. commune* [58], we utilized genome sequencing to explore the genetic differences between the two mating subtypes. Our findings suggest that subtype differentiation may be associated with deletions or mutations in one or more *Ph* genes located on the B mating-type locus. Specifically, the silencing of the *bbp2-9* and *bbp2-7* genes led to the disappearance of the G2 mating phenotypes, indicating their crucial role in determining the formation of mating subtypes. Although previous studies have shown that *Ph* genes are involved in cyclic AMP-dependent signaling pathways after recognition with *PR* genes, affecting the generation of fruiting bodies [59], our results are the first to suggest that there may be different recognition strengths of *Ph* genes, as well as the number of reciprocally recognized *Ph/PR* gene pairs may also directly influence the mating colony phenotype. While we currently lack the experimental conditions to validate B-null strains, this study lays the groundwork for understanding the role of the B mating-type locus in mating subtype differentiation [19,58]. Furthermore, the potential involvement of other genes, such as HD or PR genes [12,13], in determining mating subtypes warrants further investigation.

In natural populations of *S. commune*, environmental factors may play an essential role in driving the potential differentiation of mating subtypes. The wide distribution of *S. commune* may have become a significant external selection pressure, promoting genetic differentiation. First, different habitats may cause strains under the same mating type to undergo genetic differentiation during local adaptation. For example, under different temperature and humidity conditions, *S. commune* individuals may accumulate genomic differences, and eventually, “mating subtypes” with specific ecological adaptability will emerge within the same A/B mating type background. Second, microbial symbiotic communities may also affect the formation of mating subtypes. In different environments, the composition of the microbial communities faced by *S. commune* varies significantly. Under different symbiotic or competitive relationships, *S. commune* may form subtypes that are more adapted to specific microecological environments through local adaptation and genomic recombination. Therefore, environmental differences and biological interactions jointly drive differentiation within the same mating type *S. commune* population, laying the foundation for the long-term maintenance and ecological adaptation of mating subtypes. These phenomena provide support for how fungi maintain genetic diversity in complex environments.

## 5. Conclusions

This study advances our understanding of the tetrapolar mating system in *S. commune*. Through a detailed analysis of strain 20R-7-ZF01, we identified two distinct mating subtypes, designated as I and II. Intra- and inter-subtypes mating can produce eight mating interaction phenotypes. Intra-subtype interactions adhered to the classical G1 phenotype, while inter-subtype interactions displayed asymmetric G2 phenotypes with reduced fruiting bodies. Our analysis, covering morphological, nuclear migration, strain proportions, and genomic differences, confirms the independent nature of these subtypes and highlights their significant differences.

Mutations in pheromone genes, specifically *bbp2-9* and *bbp2-7* at the B locus, enabled us to link genetic diversity to differences in mating subtypes that influence reproductive outcomes in S. commune, as pheromone gene mutations are associated with these mating subtypes. The development of a mating subtype classification system provides a framework for understanding the complexity of the tetrapolar mating system and offers a foundation for similar studies in other fungi. This research opens new pathways for comparative research, potentially leading to a unified understanding of fungal sexual reproduction.

This work lays the foundation for future studies into the molecular mechanisms driving mating subtype-specific phenotypes, the functional roles of genes in the B locus, and the ecological and evolutionary implications of mating subtype diversity. Ultimately, it paves the way for a new era in fungal reproductive biology, with significant implications for fungal evolution and biodiversity.

## Figures and Tables

**Table 1:** Colony morphology characteristics of eight different mating interactions.

Mating Reaction	Group	Colony Morphology
A=B=	G1	Regular circular; no obvious boundary in the middle; edge smooth
	G2	Regular circular; thin hyphae on one side and dense on the other with a dividing line
A≠B≠	G1	Irregularly radial; white and dense hyphae; margin multiple fruiting bodies
	G2	Irregularly but edge smooth; sparse hyphae; margin with few fruiting bodies
A≠B=	G1	Barrage reaction separates into two halves along the medium line; white and aerial hyphae
	G2	Barrage reaction with semi-arc fence structure; white and aerial hyphae
A=B≠	G1	Flat reaction separates in half along the medium line; translucent hyphae
	G2	Flat reaction with semi-arc fence structure; translucent hyphae

**Table 2:** Interaction experiments within the same subtype strains and between different subtype strains.

Mating Subtypes		Strains	S4y	S17	S103	S180	S5y	S192	S206	S10
AxBx	I	S4y	A=B= G1							
	II	S17	A=B= G2	A=B= G1						
AxBy	I	S103	A=B≠ G1	A=B≠ G2	A=B= G1					
	II	S180	A=B≠ G2	A=B≠ G1	A=B= G2	A=B= G1				
AyBy	I	S5y	A≠B≠ G1	A≠B≠ G2	A≠B= G1	A≠B= G2	A=B= G1			
	II	S192	A≠B≠ G2	A≠B≠ G1	A≠B= G2	A≠B= G1	A=B= G2	A=B= G1		
AyBx	I	S206	A≠B= G1	A≠B= G2	A≠B≠ G1	A≠B≠ G2	A=B≠ G1	A=B≠ G2	A=B= G1	
	II	S10	A≠B= G2	A≠B= G1	A≠B≠ G2	A≠B≠ G1	A=B≠ G2	A=B≠ G1	A=B= G2	A=B= G1

Gray grid, inter-group interactions; White grid, intra-group interactions.

**Table 3:** Hyphal fusion and fluorescent nuclear migration in eight different mating interactions.

Mating Reaction	Group	Hyphal Fusion	Nuclear Migration	Nuclear Fusion	Clamp Connection
A=B=	G1	−	−	−	−
	G2				
A≠B≠	G1	+	+	+	+
	G2				
A=B≠	G1	+	+	−	−
	G2				
A≠B=	G1	+	−	−	−
	G2				

+, phenomenon occurs; −, Phenomenon does not occur.

## Data Availability

The original contributions presented in the study are included in the article/Supplementary Material, further inquiries can be directed to the corresponding authors.

## Supplementary Materials

The following supporting information can be downloaded at: https://www.mdpi.com/article/10.3390/jof11040277/s1, Figure S1: Mating combination and tetrapolar mating interactions of *Schizophyllum commune* 20R-7-ZF01 monokaryotic strains; Figure S2: Microscopic observations of *S. commune* 20R-7-ZF01; Figure S3: Tetrapolar mating interaction phenotypes of five *S. commune* dikaryotic strains; Figure S4: Fluorescence microscopy visualization of interactions between mating subtype strains; Figure S5: Green fluorescence microscopy of S4y_^Δ^*bbp2-9* and S5y_^Δ^*bbp2-7* gene-silenced strains; Figure S6: Schematic diagram illustrating mating subtype differentiation; Table S1: Primer sequences used; Table S2: Transformation plasmids for mating subtype strains; Table S3: Target fragments for gene silencing; Table S4: Homology comparison of *MatA* genes by BLASTP; Table S5: Homology comparison of *MatB* genes by BLASTP.

## Abbreviations

The following abbreviations are used in this manuscript:

G1	Colony-symmetric tetrapolar interactions
G2	Colony-asymmetric tetrapolar interactions
HD	Homeodomain
Ph	Pheromone
PR	Pheromone receptor
MAT	Mating-type
PDA	Potato Dextrose Agar
MM	Minimal medium
HAMF	Histone-Assisted Merged Fluorescence
shRNA	short hairpin RNA
NGS	Next-generation sequencing
mip	mitochondrial intermediate peptidase
β-fg	β-flanking genes
