# Supplementary material for: Evidence for the Existence of Mating Subtypes Within the Schizophyllum commune: Mating Behavior and Genetic Divergence
Source: J Fungi (Basel). 2025 Apr 1;11(4):277. doi: 10.3390/jof11040277 (PMC12028200; doi:10.3390/jof11040277)
Supplement: Supplementary file 1 [file jof-11-00277-s001.zip › jof-3514603-supplementary.pdf]

Supplementary Materials

Evidence for the Existence of Mating Subtypes Within the *Schizophyllum commune*: Mating Behavior and Genetic Divergence

Chen Chu, Dongxu Li, Linqing Gu, Sihai Yang * and Changhong Liu *

State Key Laboratory of Pharmaceutical Biotechnology, School of Life Sciences, Nanjing University, Nanjing 210023, China; mg1930112@smail.nju.edu.cn (C.C.); laolang_2012@163.com (D.L.); linqinggu01@163.com (L.G.)

* Correspondence: sihaiyang@nju.edu.cn (S.Y.); chliu@nju.edu.cn (C.L.)

S1. Supplementary Figures and Tables

S1.1. Supplementary Figures

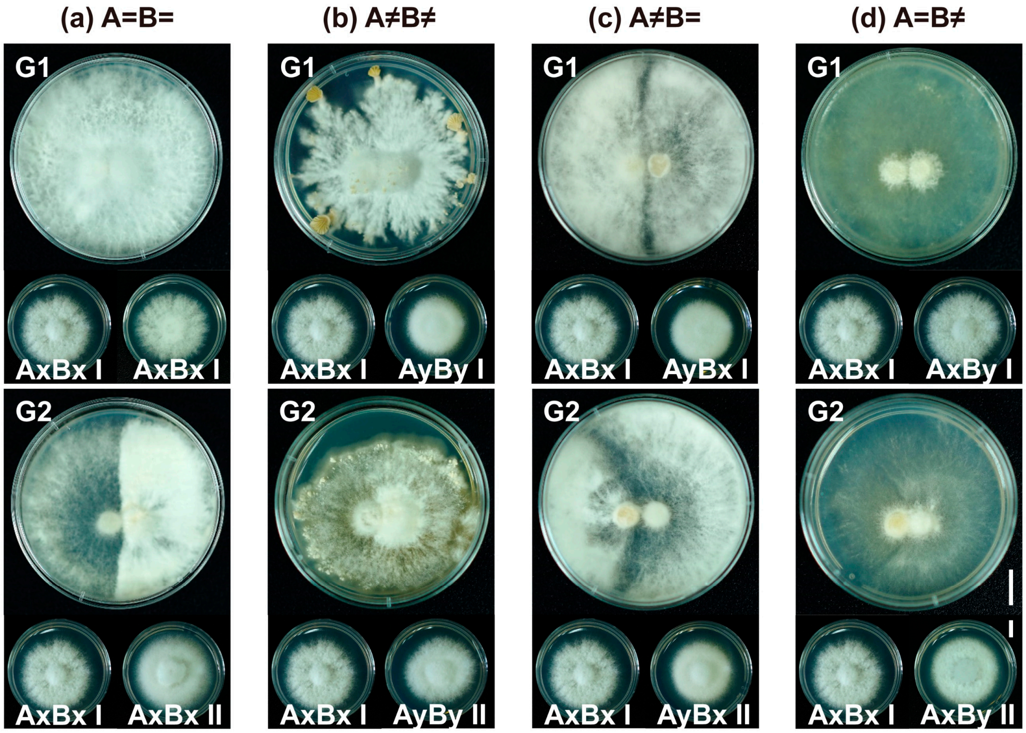

Figure S1. Mating combination and tetrapolar mating interactions of *S. commune* 20R-7-ZF01 monokaryotic strains. A set of mating combinations using the AxBx-I strain S4y is shown as an example. (a) A=B= interaction; (b) A≠B≠ interaction; (c) A=B≠ interaction; (d) A≠B= interaction. Bar = 1 cm.

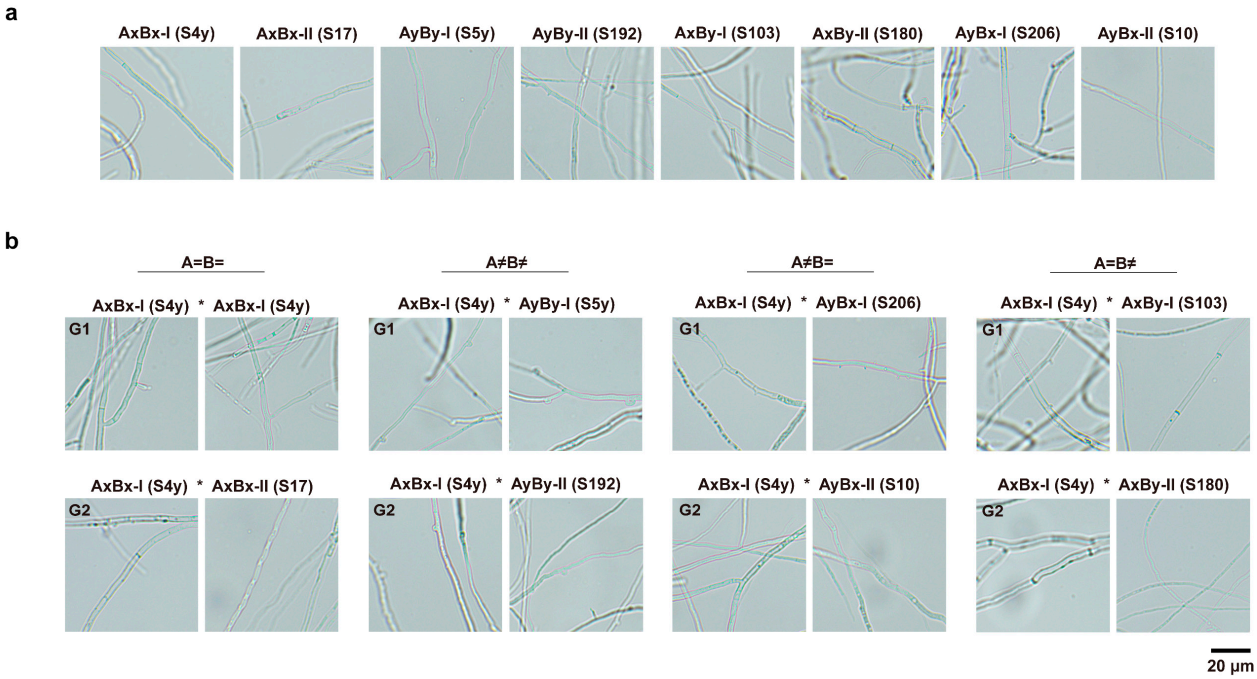

Figure S2. Microscopic observations of *S. commune* 20R-7-ZF01. Microscopic images of the tetrapolar (a) mating subtype monokaryotic strains and (b) bilateral mycelial mating interactions. The strains representing each mating subtype are indicated in parentheses. * Indicates that the strains are in a mating relationship. Bar = 20 μm.

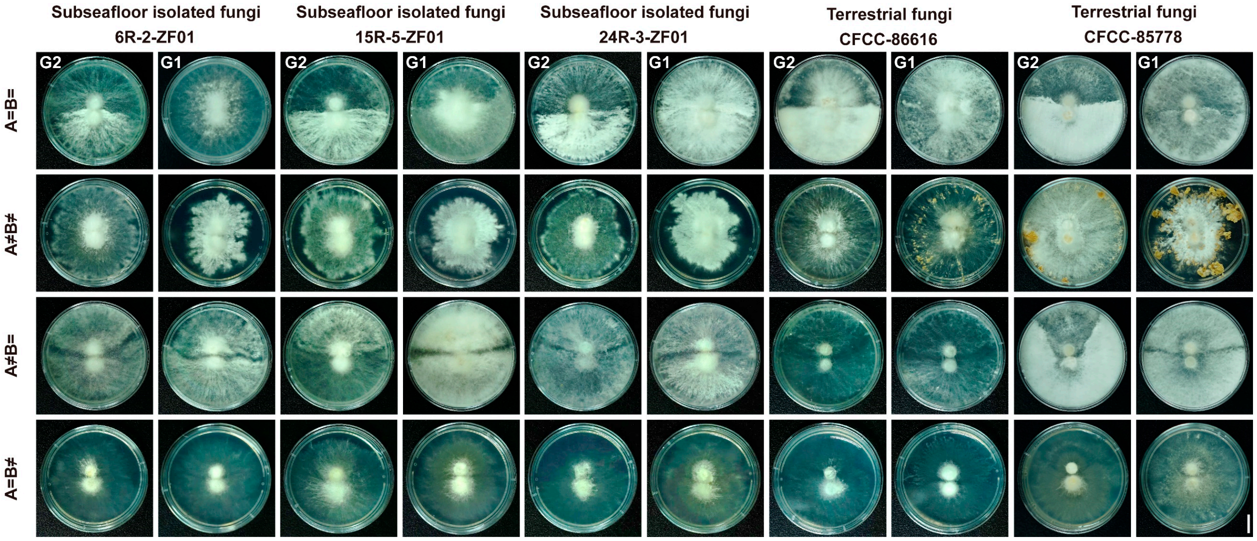

Figure S3. Tetrapolar mating interaction phenotypes of five *S. commune* dikaryotic strains. Three strains are from deep subseafloor environments (6R-2-ZF01, 15R-5-ZF01, 24R-3-ZF01) and two are from terrestrial environments (CFCC-86616, CFCC-85778). Mating interactions are classified into two groups: G1 represents symmetric phenotypes, while G2 represents asymmetric phenotypes. Bar = 1 cm.

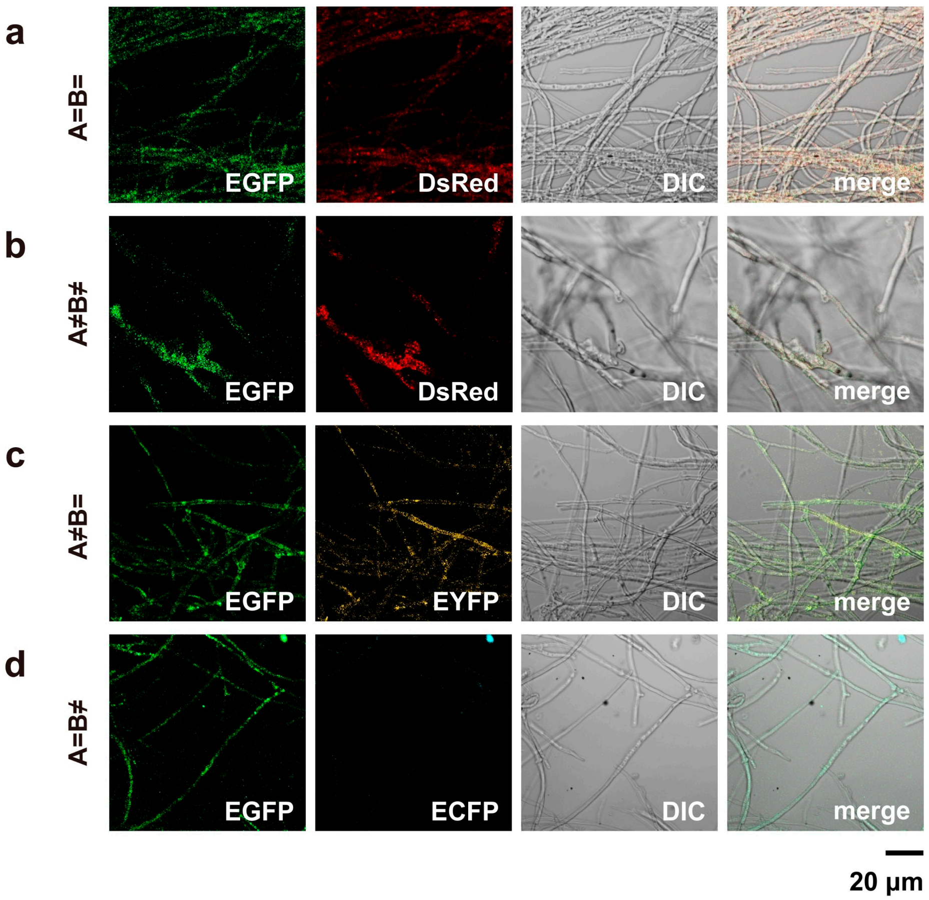

Figure S4. Fluorescence microscopy visualization of interactions between mating subtype strains. (a) A=B= interaction; (b) A≠B≠ interaction; (c) A=B≠ interaction; (d) A≠B= interaction. The micrographs show fluorescence images for EGFP, DsRed, ECFP, and EYFP, as well as DIC microscopy and merged images, as indicated. Bar = 20 μm.

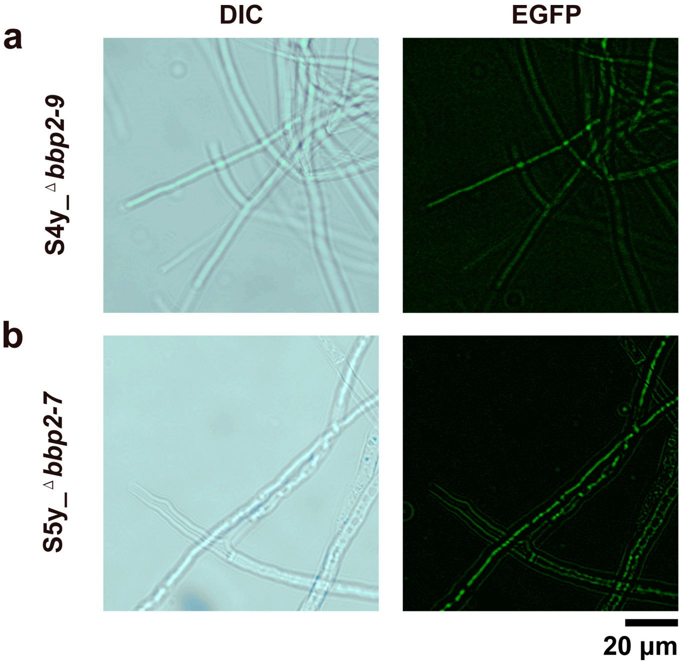

Figure S5. Green fluorescence microscopy of S4y_$^{\Delta}$*bbp2-9* and S5y_$^{\Delta}$*bbp2-7* gene-silenced strains. Bar = 20 μm.

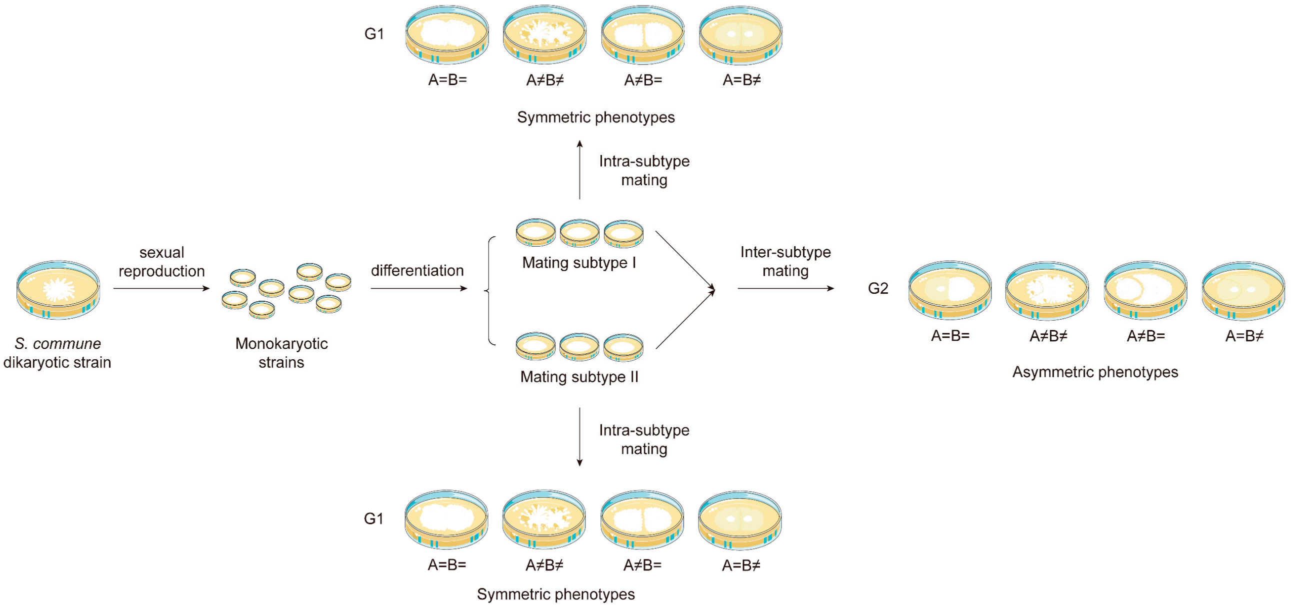

Figure S6. Schematic diagram illustrating mating subtype differentiation.

S1.2. Supplementary Tables

Table S1. Primer sequences used.

Gene	Primer	Sequence (5'-3')	Product size (bp)
H2A	H2A-QC-F	acatcaccatggATGTCTGGCAAAGTCGGC	448
	H2A-QC-R	tcaccatggcCACCTCCTGGCTGGCGTC	
DsRed1	DsRed1-F	AGGAGGTGgccATGGTGCGCTCCTC	708
	DsRed1-R	TTCTAGAGCGGCCGCTCTACAGGAACAG	
ECFP	ECFP-F	AGGAGGTGgccatggtgagcaag	732
	ECFP-R	AGAGCGGCCGCTttacttgtacagc	
EYFP	EYFP-F	AGGAGGTGgccatggtgagcaag	743
	EYFP-R	AGAGCGGCCGCTttacttgtacagc	

Table S2. Transformation plasmids for mating subtype strains.

Mating subtypes	Strains	Transformation plasmids	
AxBx I	S4y	pGH2A	pRH2A
AxBx II	S17	pGH2A	pRH2A
AyBy I	S5y	pGH2A	pRH2A
AyBy II	S192	pGH2A	pRH2A
AxBy I	S103	pCH2A	pYH2A
AyBy II	S180	pCH2A	pYH2A
AyBx I	S206	pCH2A	pYH2A
AyBx II	S10	pCH2A	pYH2A

Table S3. Target fragments for gene silencing.

Gene	Target fragments
bbp2-9	GAGCTCGTCACCGCTCGTAATA
bbp2-7	CGGACAGGGACGATGTAAATA

Table S4. Homology comparison of *MatA* genes by BLASTP.

Mating type	Gene Type		Genes	Blast_ID	Identity	Coverage	Evalue
**Ax**	**Homeodomain Genes**	**HD2**	*abr4*	XP_003037496.1	46.677	85	4.04E-138
			abv4	XP_003037495.1	42.143	28	8.03E-25
			aay4	XP_003038722.1	92.142	100	0
		**HD1**	*abs4*	XP_003038830.1	65.615	96	0
			abq4	XP_003037497.1	41.424	54	9.17E-56
			aaz4	XP_003037384.1	85.714	100	0
	**Mitochondrial Intermediate Peptidase Gene**	**MIP**	*mip*	P37932.2	98.71	100	0
	**Beta-fg Gene**	**Beta-fg**	*β-fg*	XP_003038552.1	98.624	100	1.82E-158
**Ay**	**Homeodomain Genes**	**HD2**	*abr1*	XP_003037496.1	59.41	100	0
			abv1	XP_003037495.1	31.56	44	6.41E-29
			aay1	AAB01367.1	90.556	99	0
		**HD1**	*abs1*	XP_003038830.1	43.729	50	1.87E-55
			abq1	XP_003037497.1	39.809	53	6.83E-54
	**Mitochondrial Intermediate Peptidase Gene**	**MIP**	*mip*	P37932.2	98.968	100	0
	**Beta-fg Gene**	**Beta-fg**	*β-fg*	XP_003038552.1	99.083	100	2.62E-159

Table S5. Homology comparison of *MatB* genes by BLASTP.

Mating type	Gene Type		Genes	Blast_ID	Identity	Coverage	Evalue
Bx	Pheromone Genes	Ph	*bbp2-2*	AAK58074.1	98.361	82	3.14E-40
			bbp2-1	KAI5887462.1	80	70	4.88E-32
			bbp2-9	KAI5823843.1	92.75	100	1E-39
			bbp2-6	AAR99652.1	76.471	13	5.54E-06
			bap2-3	AAR99617.1	50	44	1.81E-08
	Pheromone Receptor Genes	PR	*bbr2*	AAD35087.1	96.349	100	0
			bbr1	P78741.2	48.338	68	9.4E-102
			bar3	P56502.2	84.83	99	3.98E-147
		PR-Like	*brl1*	XP_003028241.1	71.611	100	0
			brl2	XP_003027972.1	77.373	100	0
			brl3	XP_003027973.1	98.133	100	0
By	Pheromone Genes	Ph	*bbp2-2*	AAK58074.1	41.509	60	1.71E-11
			bbp2-4	AAD35086.1	85.455	43	1.08E-32
			bbp2-8	AAR99653.1	68.235	76	1.9E-19
			bbp2-6	AAR99652.1	50.847	100	5.12E-25
			bap3-3	KAI5887475.1	63.095	100	1.08E-27
			bap3-2	AAR99650.1	87.5	70	2.22E-43
			bap3-1	KAI5887478.1	90.909	52	7.16E-26
			bbp1-2	AAB41860.1	64.701	20	1.86E-05
			bbp2-7	AAK58072.1	91.667	27	3.70E-08
	Pheromone Receptor Genes	PR	*bbr2*	AAD35087.1	65.982	82	0
			bar3	P56502.2	91.393	100	0
		PR-Like	*brl2*	XP_003027972.1	76.883	100	0
			brl3	XP_003027973.1	98.34	100	0
